# [Retracted] Sirtuin 7 promotes glioma proliferation and invasion through activation of the ERK/STAT3 signaling pathway

**DOI:** 10.3892/ol.2026.15761

**Published:** 2026-07-13

**Authors:** Pengfei Mu, Kun Liu, Qingyuan Lin, Wensheng Yang, Dan Liu, Zhen Lin, Wei Shao, Tianhai Ji

Oncol Lett 17: 1445–1452, 2019; DOI: 10.3892/ol.2018.9800

Following the publication of the above paper, it was drawn to the Editor's attention by a concerned reader that the CDK2 protein blots in Fig. 5A for the U87 and U251 cell lines on p. 1451 were strikingly similar, suggesting that the same data had been included in this figure to show the results of different experiments. In addition, for the cell invasion assay experiments shown in Fig. 4A on p. 1450, the si-Sirt7-1 and si-Sirt7-2 data panels for the U87 cell line appeared to contain an overlapping section. Upon subsequently performing an independent analysis of the data in this paper in the Editorial Office, it also came to light that: i) a pair of the β-actin blots in Fig. 2A were also featured in Fig. 2B, albeit the reported experimental conditions were different; ii) the Sirt7 blots in Fig. 2C were apparently re-used as the p-ERK blots in Fig. 5A; iii) β-actin bands in Fig. 2A doubled up as β-actin bands in Fig. 5A, where different experimental conditions were reported; iv) in Fig. 1A, the β-actin bands for patient nos. 6–8 (top panel) doubled up as the β-actin bands for patient nos. 13–15 (lower panel); and v) control β-actin data in Fig. 1A had appeared previously in a paper featuring some of the same authors in the journal *International Journal of Molecular Science*, albeit this may have been intentional based on the reported experimental conditions.

Given the large number of potentially anomalous issues identified with the abovementioned figures in this paper, the Editor of *Oncology Letters* has decided that it should be retracted from the Journal on account of a lack of confidence in the presented data. The authors were asked for an explanation to account for these concerns, but the Editorial Office did not receive a reply. The Editor apologizes to the readership for any inconvenience caused.

